# Structural Insights into Endogenous Retroviruses: Evolution and Co-Opted Functions

**DOI:** 10.3390/v18101132

**Published:** 2026-10-13

**Authors:** Cheng-An Lyu, Emma F. Harding, Zhaobo Zhang, Yao Shen, Aris Katzourakis, Peijun Zhang

**Affiliations:** 1 Chinese Academy of Medical Sciences Oxford Institute, University of Oxford, Oxford OX3 7BN, UK; cheng-an.lyu@ndm.ox.ac.uk; 2 Division of Structural Biology, Wellcome Centre for Human Genetics, University of Oxford, Oxford OX3 7BN, UK; zhaobo.zhang@st-annes.ox.ac.uk (Z.Z.); yao.shen@strubi.ox.ac.uk (Y.S.); 3 Department of Biology, University of Oxford, Oxford OX1 3EL, UK; emma.harding@biology.ox.ac.uk; 4 Diamond Light Source, Harwell Science and Innovation Campus, Didcot OX11 0DE, UK

**Keywords:** endogenous retroviruses, structural biology, retroviral evolution, HERV-K, capsid assembly, retroviral enzymes, envelope glycoproteins, syncytins, viral domestication

## Abstract

Endogenous retroviruses (ERVs) are the molecular remnants of ancient retroviral infections and constitute a substantial fraction of many vertebrate genomes. Although most ERV loci have lost coding capacity and are epigenetically silenced, some remain biologically relevant because they have been co-opted for host physiology. Recent advances in structural biology are beginning to clarify how these elements have evolved from infectious retroviruses into genomic components with diverse molecular and cellular functions. In this review, we organise current structural insights by retroviral functional modules: group-specific antigen (Gag), polymerase (Pol) and envelope (Env). In the Gag section, we describe capsid-related assemblies of human endogenous retrovirus K (HERV-K), including mature-like CA assemblies reconstituted in vitro and immature lattices resolved in intact virus-like particles, as well as structural studies of the endogenous lentiviruses RELIK and PSIV. In the Pol section, we discuss HERV-K reverse transcriptase, biochemical studies of protease, and emerging structural evidence for integrase. In the Env section, we review prefusion and postfusion structures of HERV-K Env and receptor-bound structures of domesticated Env proteins, including syncytin-1, syncytin-2 and suppressyn. Together, these studies show how structural approaches can connect ERV evolution with molecular function, while also defining major unresolved questions regarding conformational dynamics, host interactions, and physiological relevance.

## 1. ERV Classification and Evolution

Ancient infections of the germline resulted in the integration of viral sequences into host chromosomes, where they are inherited as endogenous viral elements (EVEs) [1]. Some EVEs persist over evolutionary timescales and may subsequently be co-opted for host functions [2]. The most abundant EVEs are derived from members of the *Retroviridae*, reverse-transcribing RNA viruses that convert their genomic RNA into DNA and integrate it into the host genome as part of their replication cycle [3]. These repeated germline invasions have left thousands of viral gene fragments in modern animal genomes, known as endogenous retroviruses (ERVs). Canonical ERV proviruses retain the basic retroviral genomic organisation of *gag*, *pol*, and *env*, flanked by 5′ and 3′ long terminal repeats (LTRs). The LTRs contain promoter and enhancer elements that drive transcription, as well as sequence motifs required for reverse transcription and integration. Within this organisation, *gag* encodes the primary structural polyprotein that builds the particle. Gag is typically processed into matrix (MA), which targets assembly to cell membranes, capsid (CA), which forms the protein lattice of immature and mature cores, and nucleocapsid (NC), which binds and packages viral RNA and helps coordinate early steps of reverse transcription. The enzymatic coding regions, *pro* and/or *pol* depending on the retrovirus, encode protease (PR), which drives maturation by cleaving Gag and Pol, reverse transcriptase (RT), which synthesises viral DNA from RNA templates, and its associated RNase H domain, which degrades the RNA strand of RNA–DNA hybrids, and integrase (IN), which inserts the viral DNA into the host genome. Finally, *env* encodes the viral entry machine. Env is synthesised as a precursor that is cleaved into a receptor-binding surface subunit (SU) and a membrane-anchored transmembrane subunit (TM), which together mediate attachment and membrane fusion through a class I fusion mechanism.

The genomic abundance of ERVs varies across species. It is estimated that human endogenous retroviruses (HERVs) constitute approximately 8% of the human genome [4,5]. Surveys of rodent and marsupial genomes also reveal substantial variation [6,7,8,9,10]. For example, ERV-derived sequences comprise 1.55% of the Tasmanian devil genome [9] and approximately 10% of the grey short-tailed opossum genome [11], although estimates depend on annotation methods and the classes of remnants included. In birds, ERVs are generally less prevalent, consistent with their comparatively compact genomes [12,13]. In fish, amphibians, and reptiles, ERV abundance is highly heterogeneous and often species-specific [14,15,16]. In invertebrates, bona fide endogenous retroviruses are less clearly defined than in vertebrates [17]; instead, most related sequences are described as retrovirus-like LTR retrotransposons, including gypsy/errantivirus- and BEL/Pao-like elements, which are widespread and often show strong lineage-specific variation in abundance [18,19,20]. Such variation reflects differences in the historical frequency of germline invasion, lineage-specific amplification and decay of ERV families, the efficiency of host silencing and deletion mechanisms, and broader genome-size and transposable-element dynamics [14].

ERV classification varies across schemes, partly because many ERV loci are highly degraded, their lineages are extinct, and recombination and mosaicism can obscure their evolutionary relationships [14,21,22]. Repeat-annotation resources such as Repbase and Dfam use sequence-based categories, including ERV1, ERV2/ERVK and ERV3/ERVL [23,24]. These correspond broadly to the traditional Classes I, II and III, which group gamma/epsilon-, beta/alpha/lenti/delta-, and spuma-like retroviruses, respectively [14,15,21]. Repbase additionally recognises ERV4, a distinct group described in non-mammalian vertebrates [23]. These repeat-annotation categories should be distinguished from formal viral taxonomy.

**Figure 1:**
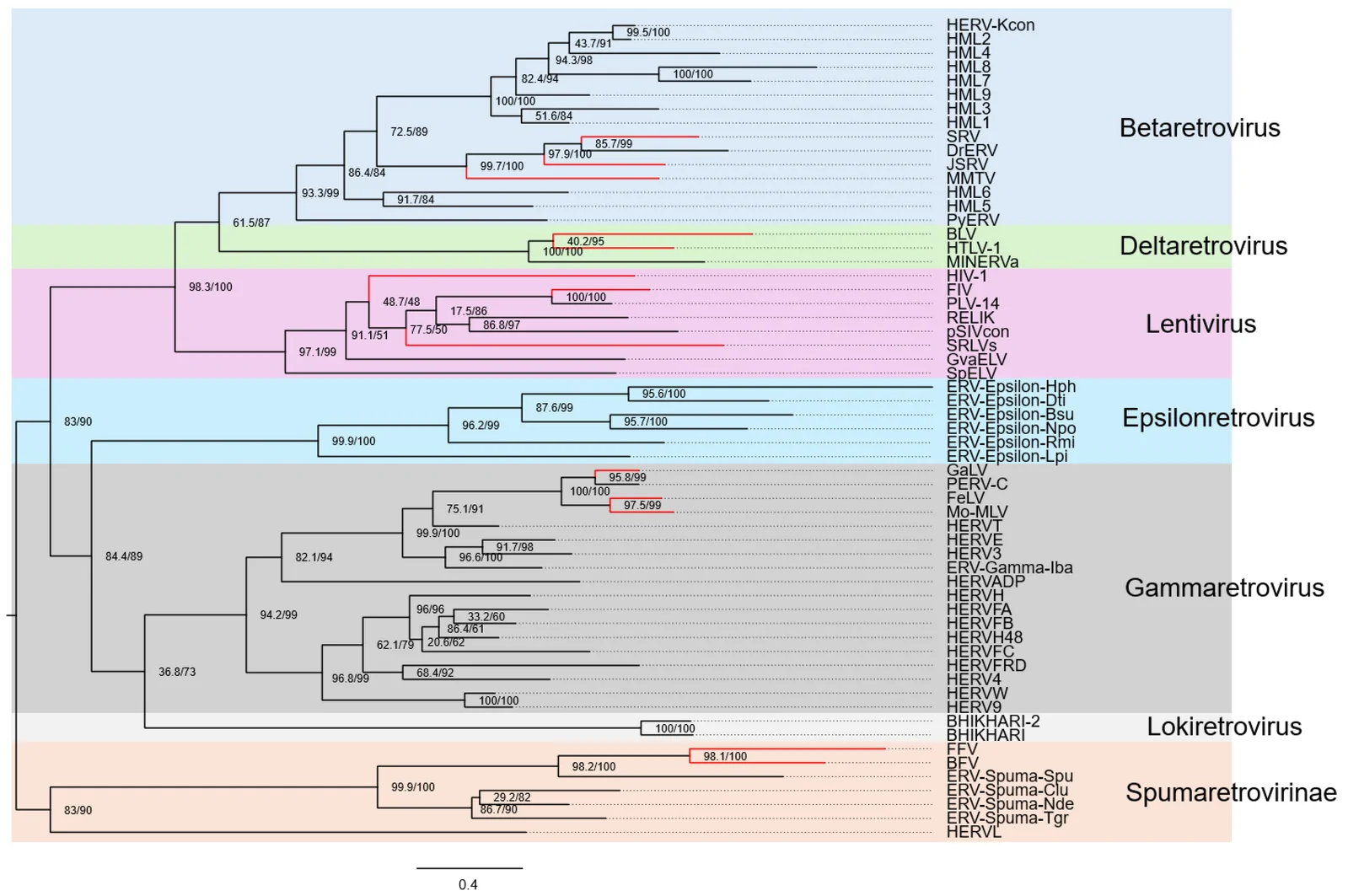
Gag-based phylogenetic tree of representative ERVs and retroviruses. A maximum-likelihood tree was inferred from Gag. Sequences were aligned with MAFFT v7.526 (L-INS-i) and trimmed with trimAl v1.5.rev0 (-gappyout), retaining 298 of 1812 columns (7.5% gaps; 20.5% mean pairwise identity; 296 parsimony-informative sites, no constant sites). Sequences were analysed with IQ-TREE 3.0.1 under LG+F+R4, selected by ModelFinder (BIC), with 1000 ultrafast bootstrap replicates, and 1000 SH-aLRT replicates; branches with SH-aLRT ≥ 80 and bootstrap ≥ 95 are considered robust (32 of 56 internal branches). The tree is rooted on the class III (spuma-like) sequences by convention. Shaded regions indicate established ERV/retroviral lineage assignments, independent of the phylogeny; BHIKHARI is shaded separately because its position in this Gag tree is unresolved; the Lokiretrovirus label follows its published classification. Red terminal branches indicate exogenous retroviruses, whereas black terminal branches indicate endogenous retroviral sequences. Internal branches are shown in black. Dotted lines connect tips to aligned labels and do not represent branch length. Reference sequences are listed in Supplementary Table S1 [16,21,27,30,31,32,33,34].

**Figure 2:**
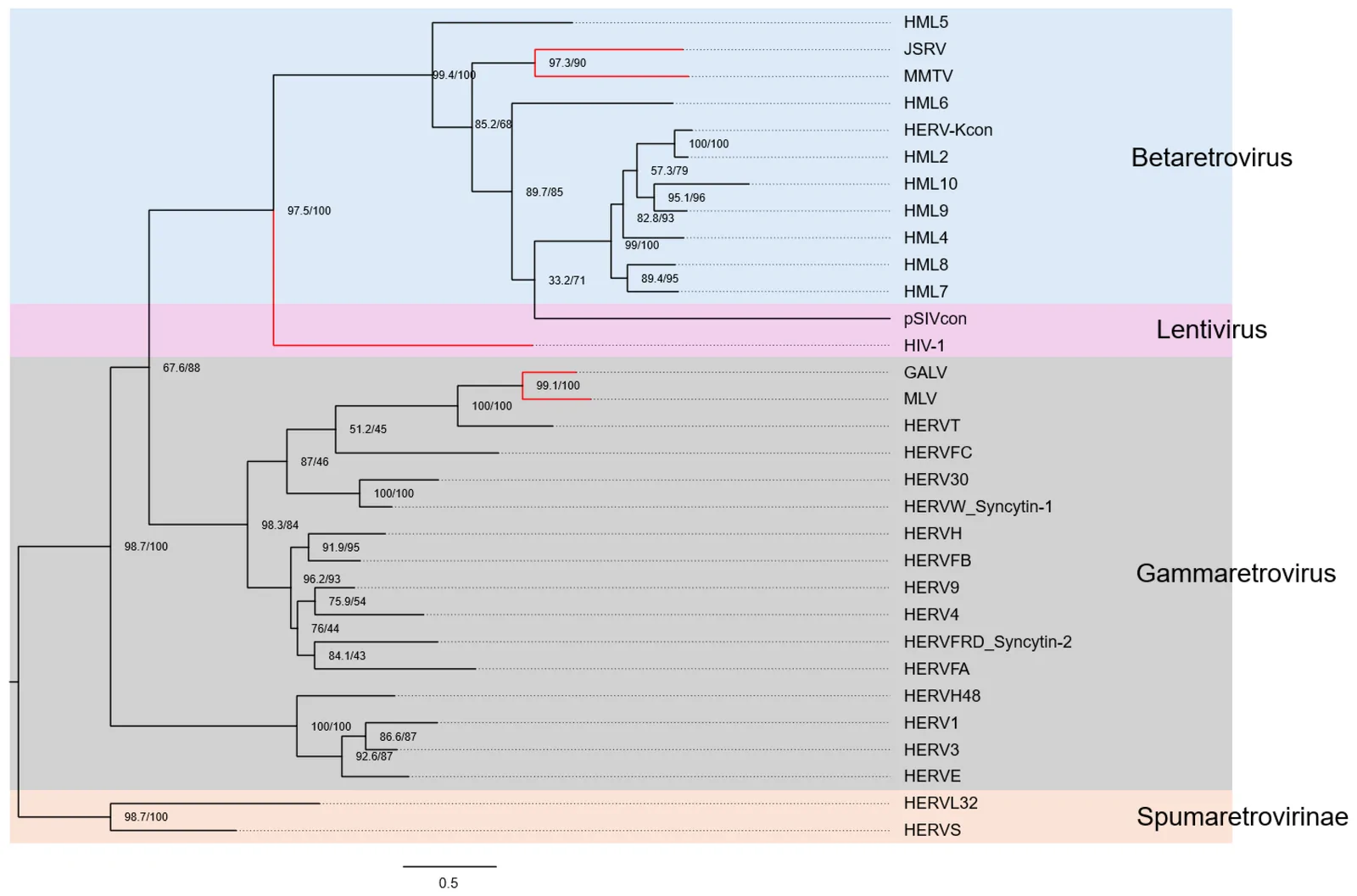
Env-based phylogenetic tree of representative ERVs and retroviruses. A maximum-likelihood tree of 31 Env sequences. Sequences were aligned with MAFFT v7.526 (L-INS-i) and trimmed with trimAl v1.5.rev0 (-gappyout), retaining 331 of 1675 columns (6.2% gaps; 21.7% mean pairwise identity; 325 parsimony-informative sites, none constant). Sequences were analysed with IQ-TREE 3.0.1 under WAG + F + G4, selected by ModelFinder (BIC), with 1000 SH-aLRT and 1000 ultrafast bootstrap replicates; values at nodes are SH-aLRT/ultrafast bootstrap. Branches were considered robust at SH-aLRT ≥ 80 together with bootstrap ≥ 95, a threshold met by 12 of 28 internal branches. The tree is displayed with the HERVS/HERVL32 branch (98.7/100) as a conventional root. Their class III assignment refers to their genomic backgrounds and does not establish an Env-specific outgroup. Shading denotes established lineage assignments and does not imply monophyly: the betaretrovirus-related sequences form a larger clade with the lentiviral sequences (97.5/100), within which pSIVcon falls among the HMLs, and the gammaretroviral sequences are recovered as two separate groups (98.3/84 and 100/100). Red terminal branches indicate exogenous retroviruses, whereas black terminal branches indicate endogenous retroviral sequences. Internal branches are shown in black. Dotted lines connect tips to aligned labels and do not represent branch length. Reference sequences are listed in Supplementary Table S2 [21,30,34].

Retroviral classification is primarily based on the polymerase gene because it is the most conserved region of the retroviral genome and therefore the easiest to relate between divergent viruses. Consistent with this, Pol-based phylogenies have long provided the main framework for retroviral and ERV classification, and comprehensive Pol-based phylogenetic trees have already been reconstructed across vertebrate genomes [25]. By comparison, Gag- and Env-based evolutionary relationships remain less systematically explored, despite the fact that *gag*- and *env*-derived genes include many of the best-known examples of ERV co-option. Because these proteins underlie functions such as particle assembly, membrane fusion, and host defence, Gag- and Env-based phylogenies may provide additional insight into the origins and diversification of co-opted ERV functions. Accordingly, in this review we also focus on the structural and biological roles of ERV-derived Gag- and Env-related proteins and place them in evolutionary context using phylogenetic trees reconstructed from conserved regions of Gag and Env (Figure 1 and Figure 2).

Paleovirology reveals broad, long-term patterns of retroviral infection across vertebrate evolution. For example, the deltaretrovirus- and lentivirus-related members of class II have so far been identified only in mammals, consistent with the idea that these lineages are more recent additions to retroviral diversity [26,27]. By contrast, more basal vertebrates, including fish and amphibians, are enriched for class I epsilon- and gamma-like ERVs, supporting an older evolutionary origin for these groups [28,29]. Host distribution alone does not establish the relative ages of these viral lineages. Within humans, HERV sequences span several major retroviral lineages, reflecting repeated endogenisation events from diverse ancestral retroviruses over evolutionary time. The Gag-based phylogeny resolves five retroviral assemblages at or above our robustness threshold: class I gamma-like (94.2/99) and epsilon-like ERVs (99.9/100), class II betaretrovirus-related ERVs (93.3/99), deltaretrovirus-related sequences (100/100) and lentivirus-related sequences (97.1/99). Only the class III spuma-like group falls below it (83/90) (Figure 1). Within this framework, the HERV-K HML families cluster within the class II betaretrovirus-related group, whereas most other human ERV families fall within class I lineages. The tree also places human spuma-like HERV elements within the broader class III assemblage, indicating that foamy virus-like ancestors contributed at least *gag*-related sequences to the human genome.

The Env-based tree provides a complementary perspective that is especially relevant for interpreting co-opted ERV functions (Figure 2). Deep sequence divergence limits interpretation of the Env tree, as only 12 of 28 internal branches meet the stated support thresholds, compared with 32 of 56 for Gag. The recovered groupings provide context for discussing host co-option, while poorly supported relationships remain unresolved. In particular, HERV-K HML Env sequences again group within a betaretrovirus-related clade, whereas several other HERV Env families, including those related to syncytin genes, fall within class I gamma-like assemblages. This pattern is consistent with the idea that distinct functional outcomes of ERV domestication emerged from different retroviral lineages. Notably, Env-derived genes have been repeatedly co-opted for host functions such as trophoblast fusion and antiviral receptor interference [35].

Following germline invasion and vertical transmission, most ERV loci accumulate inactivating mutations, including nonsense substitutions, frameshifts, and recombination-mediated deletions, thereby losing their capacity for replication and reinfection [3,36]. In addition to damage accumulated after integration, APOBEC3 restriction factors can introduce mutations into viral cDNA during reverse transcription, leaving hypermutation signatures in the resulting insertions [37,38]. Alternatively, some ERVs lose their envelope genes entirely and transition to a purely retro-transpositional replication strategy, effectively becoming genomic parasites [9,39,40]. Over evolutionary time, some non-deleterious insertions become fixed in host populations and are epigenetically silenced [14,36,41]. Exceptions arise when (i) integration is evolutionarily recent and coding potential remains only partially disrupted [8,42], or (ii) viral sequences have been co-opted for host biological functions [14,43]. The first category is exemplified by HERV-K (HML-2), one of the most recently integrated and coding-intact HERV lineages in the human genome, in which some loci retain substantial coding potential and are transcribed in human cells [5,44], and reconstructed consensus sequences can generate viral proteins and virus-like particles [34,45,46]. The second category includes domesticated retroviral or retroelement-derived proteins, including neuronal Arc proteins and envelope-derived syncytins in mammals and some reptiles [47,48,49,50,51,52,53,54]. These examples illustrate a long-term evolutionary transition from ancestral infectious retroviruses to fixed genomic elements that are largely replication-defective, with a subset recruited for host biological processes.

## 2. ERVs Through a Structural Lens

Multiscale structural studies can connect ERV sequence history with the organisation of proteins and particles [55]. Single-particle cryo-EM can determine structures of intact virions and particles, including their internal components, as well as isolated complexes. Cryo-electron tomography (cryo-ET) is particularly valuable for visualising assemblies in their cellular context and for resolving the three-dimensional organisation of individual virions and particles; subtomogram averaging can improve the resolution of repeated assemblies. Crystallography and solution NMR provide complementary information on molecular folds, interactions and conformational dynamics. Structural observations at these different scales guide mechanistic hypotheses, whose physiological relevance should be evaluated through functional experiments. ERV discovery and annotation remain difficult because coding-sequence degradation and divergence can obscure reconstructable proteins [56]. Reconstruction can nevertheless recover experimentally testable proteins, as also illustrated outside retroviruses by studies of endogenous parvovirus capsids [57]. For ERVs, the key interpretive distinction is between a naturally encoded protein, a reconstructed consensus and an engineered construct. Each can reveal molecular principles, but their relationship to a particular endogenous locus must be established separately.

Machine learning has expanded the structural toolkit available for ERV research. AlphaFold and related approaches underpin viral structure resources such as BFVD and Viro3D, as well as proteome-wide analyses of viral protein families [58,59,60], enabling fold-based searches alongside sequence comparisons. Structure-guided analyses can inform evolutionary relationships, as illustrated by work on *Flaviviridae* [61], and can help identify candidate functions for highly divergent viral proteins [61,62]. However, a predicted fold is a hypothesis about molecular architecture. It does not establish expression, enzymatic activity, oligomeric state or physiological function. Similar folds also require evaluation against sequence, genomic and phylogenetic evidence before they are interpreted as evidence of common ancestry.

Experimental and computational approaches are therefore most informative when used together. Predictions can guide construct design and identify candidate interfaces; experimental structures can test those models and reveal features that predictions do not reliably capture. Functional assays are then needed to determine whether a proposed interface, conformational change or catalytic site contributes to the biological process of interest. Throughout the following sections, we distinguish structural observations from functional inference and consider how construct design and experimental context constrain interpretation.

## 3. Endogenous Capsid Remnants and Assemblies

Retroviral Gag proteins build the structural framework of virions by coordinating membrane binding, genome packaging, and particle assembly [63]. Canonically, Gag multimerises into an immature hexameric lattice beneath the viral membrane, with MA forming a membrane-associated layer, CA constituting the main lattice, and NC binding the genomic RNA [64,65]. During maturation, proteolytic cleavage of Gag triggers a major CA rearrangement that produces the mature capsid core. In vitro, purified CA from several retroviruses can self-assemble into fullerene-like lattices composed of hexamers and pentamers, providing a useful model for the geometric principles that govern mature capsid formation, as shown for HIV [66], Rous sarcoma virus (RSV) [67] and HERV-K [68]. In intact virions and virus-like particles, these same fullerene principles give rise to mature cores with diverse morphologies, including the conical capsids of HIV-1 [69] and the more polyhedral architectures observed in murine leukaemia virus (MLV) [70] and RSV [71]. The structural characterisation of mature capsids has long proven challenging due to their pleomorphic, diverse, and often metastable nature. Advancements in the structural analysis of exogenous retroviruses have been facilitated by enhanced methodologies, including X-ray crystallography, single-particle cryo-EM, and in situ cryo-ET [55,72], alongside refined sample preparation techniques, notably biochemical reconstitution strategies employing assembly cofactors such as inositol hexaphosphate (IP6) to stabilise retroviral capsid lattices [66]. These advances have made it possible to resolve mature capsid architectures in multiple retroviral systems.

### 3.1. HERV-K Gag/CA Structures and Implications for Maturation

**Figure 3:**
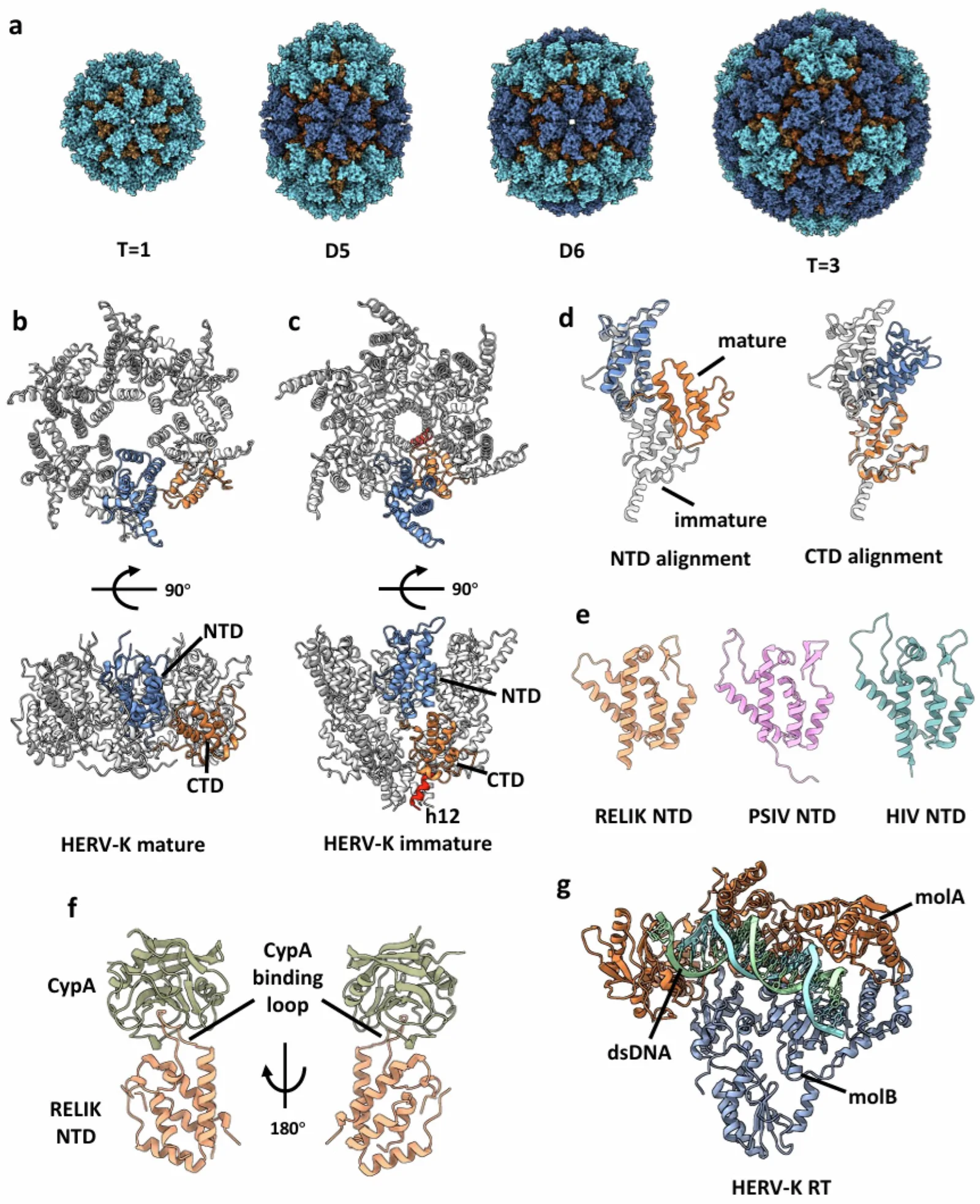
Structural landscapes of endogenous *gag*- and *pol*-derived proteins. (**a**) Surface representations of the in vitro assembled HERV-K HML-2 capsids. T = 1 particle: 60 monomers arranged as 12 pentamers (PDB: 6SSJ). D5 particle: 90 monomers arranged as 12 pentamers and 5 hexamers (PDB: 6SSK). D6 particle: 108 monomers arranged as 12 pentamers and 8 hexamers (PDB: 6SSL). T = 3 particle: 180 monomers arranged as 12 pentamers and 20 hexamers (PDB: 6SSM). Pentamer NTDs, cyan; hexamer NTDs, blue; CTDs, orange. (**b**) Mature-like HERV-K capsid hexamer reconstructed from consensus CA assembled in vitro (PDB: 6SSM). NTD: blue; CTD: orange. Curved arrow indicates a 90° rotation. (**c**) Immature HERV-K virus-like particle lattice determined by cryo-electron tomography and subtomogram averaging (PDB: 8C9M). NTD: blue; CTD: orange; h12: red. Curved arrow indicates a 90° rotation. (**d**) Comparison of mature-like (NTD blue, CTD orange) and immature (grey) HERV-K capsid protein conformations, showing differences in interdomain arrangement (PDB: 6SSM, mature-like; 8C9M, immature). (**e**) Structural comparison of endogenous lentiviral capsid N-terminal domains from RELIK (orange, PDB: 2XGU) and PSIV (pink, PDB: 2XGV) with HIV-1 CA NTD (cyan, PDB: 5MCX), illustrating conservation of the lentiviral CA fold despite deep evolutionary divergence. (**f**) Crystal structure of the RELIK CA NTD (orange) in complex with cyclophilin A (olive; complex PDB: 2XGY), highlighting the cyclophilin binding interface on the CA CypA binding loop. Curved arrow indicates a 180° rotation. (**g**) Structure of HERV-K reverse transcriptase bound to dsDNA and an incoming nucleotide (PDB: 7SR6), showing its asymmetric dimeric organisation. molA: orange; molB: blue; dsDNA: cyan.

Acton et al. [68] characterised mature-like HERV-K (HML-2) CA assemblies reconstituted in vitro from a reconstructed consensus sequence. Single-particle cryo-EM resolved four classes of closed, fullerene-like assemblies at 2.7–4.3 Å (Figure 3a). The dominant T = 1 shell contains 12 pentamers; D5, D6 and T = 3 particles combine pentamers with adaptable hexamers (Figure 3a,b). Complementary crystallographic and NMR structures confirmed conservation of the orthoretroviral CA fold, comprising an N-terminal domain (NTD) and a C-terminal domain (CTD). The assembly structures demonstrate how a conserved CA scaffold accommodates different curvatures. However, consensus reconstruction combines sequence information across loci, and assembly was induced at high salt concentrations (>1 M NaCl). The observed polymorphism therefore defines structures accessible under these experimental conditions. It neither establishes the distribution of native core morphologies nor distinguishes biological heterogeneity from effects of reconstitution. Comparisons with particles produced from defined endogenous loci are needed to determine which features extend to native capsids.

Krebs et al. [65] used cryo-ET and subtomogram averaging to resolve the immature HERV-K CA lattice at 3.2 Å in intact virus-like particles (VLPs) released from HEK293T cells expressing consensus HERV-K Gag. This preserves the relationship between the lattice and membrane, although it remains a reconstructed expression system. The greater membrane-to-CA spacing than in HIV-1 is consistent with additional Gag segments, including p15 and SP1, and shows how a conserved CA lattice can be accommodated within a different Gag architecture. The immature lattice contains CA hexamers organised around a central six-helix bundle formed by h12 (Figure 3c). The h12 helix in Krebs et al. corresponds to the terminal CTD helix α11 in Acton et al. The different numbering reflects the assignment of seven versus six helices to the NTD, respectively. Density coordinated by lysine rings is consistent with an IP6-like cofactor. Comparisons with other immature retroviral lattices reveal shared and lineage-specific architectural features [73,74,75]. The structure places h4 at the trimer interface and h9 at the dimer interface between adjacent hexamers. Molecular dynamics simulations assessed the persistence and flexibility of individual contacts. Mutational testing was focused on the trimer interface: Y66A, E69A, K85A, D90A and R97A abolished detectable VLP production, whereas R100A, Q118A, S121A and T122A retained it. These results support a contribution of selected trimer-interface residues to particle production. They do not isolate the energetic contribution of each contact or establish equivalent mutational requirements at the dimer interface.

Together, these studies show that HERV-K CA can retain its domain folds while changing its interdomain arrangement and intermolecular contacts. Pentamer incorporation closes the reconstructed mature-like shells [68], whereas the immature lattice centres on hexamers and a six-helix bundle. This comparison is consistent with conserved principles of retroviral maturation [72], but it does not directly visualise the transition between states (Figure 3d). A useful next step is to compare defined locus variants in matched immature and mature particles. Such experiments could test whether changes in the interdomain linker, terminal helix or cofactor-binding region alter assembly and maturation, rather than assuming that consensus behaviour applies to every locus.

### 3.2. Endogenous Lentiviruses RELIK and PSIV and an Ancient CA Cyclophilin Interface

The endogenous lentiviruses RELIK, from rabbits, and PSIV, from lemurs, offer a complementary test of functional conservation. Goldstone et al. [76] reconstructed their capsid sequences and introduced them into equine infectious anaemia virus (EIAV), retaining the EIAV protease-cleavage context. The resulting chimaeras supported infection, including in non-dividing cells. This demonstrates compatibility with an extant lentiviral replication system, but does not reconstruct the complete ancestral viruses or establish their original host range.

Crystal structures of the RELIK and PSIV CA NTDs, together with a RELIK CA–cyclophilin A (CypA) complex, revealed a conserved lentiviral fold and an ancient CypA-binding interface (Figure 3e,f). The RELIK complex resembles the HIV-1 binding mode while retaining local differences in interfaces. Susceptibility of the chimaeras to restriction by the TRIM5–cyclophilin A fusion protein (TRIMCyp) links this structural conservation to a functional phenotype [76]. The combined structural and infection evidence is stronger than fold similarity alone. Nevertheless, the isolated NTD structures do not define the complete ancestral capsid lattice, and the EIAV background may compensate for properties that would differ in the original viruses.

### 3.3. Gag-Derived Host Functions in Arc and BHIKHARI

Arc provides an experimentally established comparison for Gag domestication. Derived from Ty3/gypsy retrotransposons, it is a related retroelement example rather than a canonical ERV protein [52,53]. Cryo-EM structures of *Drosophila* dArc1 and dArc2 revealed assembled capsids at 2.8 and 3.7 Å, respectively [77]. Together with evidence for Arc1-mediated RNA transfer [53], these findings show how an ancestral assembly scaffold can be retained in a cellular context. The purified capsids establish assembly capacity, but do not imply that every Arc homologue forms an identical particle or uses the same regulatory mechanism in vivo.

BHIKHARI provides an emerging example involving recently active ERV families. Genetic experiments implicate zebrafish Bik-1 Gag in mesoderm development and Bik-2 Gag in neural crest development [78]. Their proposed CA-like architecture currently rests on computational predictions, which do not establish capsid formation or an assembly-dependent mechanism. These developmental functions illustrate host dependence on products of recently active elements, rather than demonstrating fixation as conventional host genes. The phenotypes motivate structural investigation but do not establish that BHIKHARI proteins use the same assembly mechanism as viral Gag or Arc.

## 4. Structures and Unresolved Questions in ERV Enzymology

Retroviral enzymes mediate polyprotein processing, reverse transcription and integration. Protease (PR) cleaves viral polyproteins during particle maturation. RT synthesises DNA from an RNA template, while its associated RNase H domain degrades the RNA strand of RNA–DNA hybrids [79,80,81]. Integrase (IN) catalyses insertion of the resulting viral DNA into the host genome. RT sequence conservation also provides a basis for retroviral evolutionary analysis [82]. The following sections examine structural and biochemical studies of HERV-K RT, PR and IN, with particular attention to substrate recognition and inhibitor interactions.

### 4.1. HERV-K Reverse Transcriptase

Baldwin et al. determined a 2.6 Å structure of HERV-K RT bound to dsDNA and an incoming nucleotide [83]. It forms an asymmetric homodimer: the identical chains, molA and molB, adopt different conformations (Figure 3g). This resembles the functional asymmetry of HIV-1 RT without requiring the same p66/p51 proteolytic processing. The RNase H domain of molB was disordered rather than proteolytically absent. The structure establishes a substrate-bound architecture, but does not resolve the full catalytic cycle or demonstrate that every endogenous RT variant adopts the same state.

Biochemical assays showed inhibition by nucleoside analogue triphosphates, with reduced potency relative to HIV-1 RT, particularly for lamivudine triphosphate (3TC-TP). Nevirapine and efavirenz did not inhibit HERV-K RT under the tested conditions [83]. These results show that conservation of the overall fold does not ensure conservation of drug sensitivity; they do not exclude other allosteric inhibitors. The immediate value of the structure is to guide selective probes. Demonstrating that RT activity causes a disease-associated phenotype will require target engagement and functional experiments beyond inhibition of the purified enzyme. Whether these structural and inhibitor-response properties extend to RTs from other ERV families, including HERV-H and HERV-W, requires direct experimental comparison.

### 4.2. Protease and Integrase

HERV-K10 PR has been studied biochemically, and inhibitor interactions were interpreted using a homology model based on HIV-1 PR [84]. Earlier work on HERV-K IN similarly combined drug-sensitivity experiments with comparative modelling of its active site [85]. These studies provide functional constraints, but their models should be distinguished from experimentally determined structures.

Direct structural evidence for HERV-K IN is reported in a June 2026 preprint by Barrena-Martin et al. [86]. Using recombinant full-length HERV-K108 IN and short DNA substrates, the authors resolved tetrameric intasomes, including a 3.02 Å strand-transfer complex assembled on pre-joined DNA oligonucleotides that mimic the integration product. Outer-subunit CTDs contribute to the synaptic interface within this compact assembly, differing from the octameric mouse mammary tumour virus (MMTV) intasome used for comparison. The structure defines a product-like architecture; the covalent junctions were supplied in the substrate rather than generated during structure determination.

Raltegravir-bound complexes were resolved in open and closed conformations at 2.89 and 3.13 Å, respectively [86]. The inhibitor coordinates active-site Mg^2+^ ions and displaces the terminal viral-DNA nucleotide; the closed conformation additionally narrows the target-DNA-binding cleft. Separate PCR-based assays and sequencing of LTR–target junctions support strand-transfer activity on a 1-kb DNA substrate, and the PCR assay supports raltegravir inhibition. These experiments extend the earlier modelling work to direct structural observations. However, the proposed closed-state role in the catalytic cycle remains a mechanistic hypothesis, and the reconstituted system does not establish cellular integration behaviour or therapeutic efficacy. The study has not yet been peer-reviewed.

## 5. Env-Derived Fusogens and Receptor Engagement

Retroviral Env proteins mediate host cell entry [87]. They are class I fusion proteins produced as precursors that are cleaved into a receptor-binding surface subunit (SU) and a membrane-anchored transmembrane subunit (TM) [87,88]. Activation exposes the fusion peptide or loop, allowing insertion into the target membrane and refolding into a postfusion trimer of hairpins, commonly a six-helix bundle (6HB) [87,89]. ERV genome surveys reveal diverse lineages with variable retention of *env* [90]. Structural comparison can help distinguish conserved fusion machinery from divergent receptor-binding surfaces. For example, syncytin TM structures retain the class I hairpin architecture despite substantial sequence divergence [91,92]. This illustrates the value of structure for recognising a shared mechanism, without implying that structural similarity alone resolves evolutionary relationships or identifies neutralising epitopes.

### 5.1. HERV-K Env Structures and Antibody Targeting

HERV-K Env expression has been associated with cancer, neurological disorders, viral infections and ageing [4,45,47,93,94]. HML-2 transcripts are also widespread in normal tissues, including the cerebellum, pituitary, testis and thyroid [44]. Transcript abundance alone does not establish Env protein production, its conformation or a causal role in disease. Structural studies provide a framework for testing these distinctions by defining conformational states and antibody-binding surfaces.

**Figure 4:**
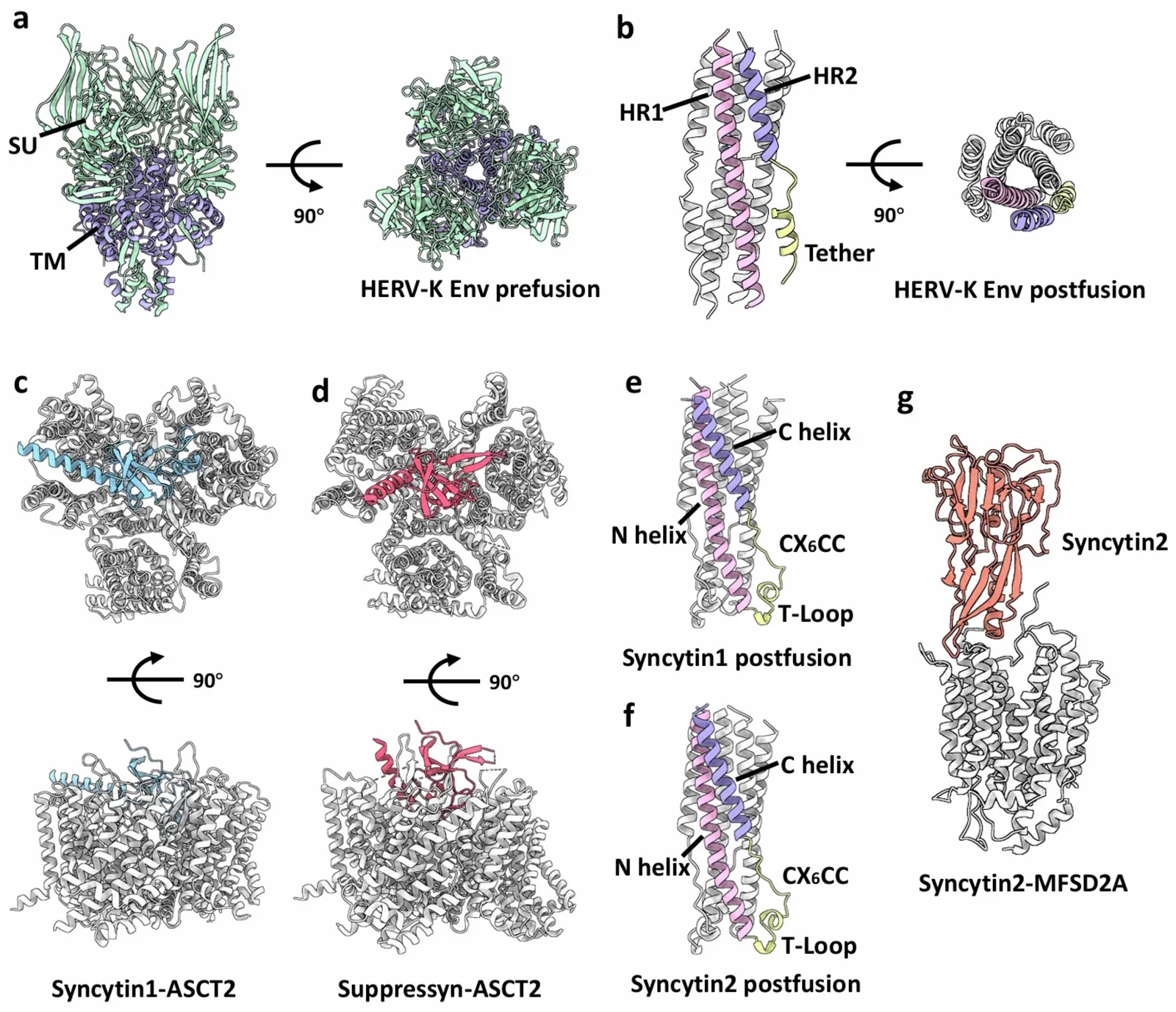
Structural landscapes of endogenous *env*-derived proteins. (**a**) Prefusion structure of the HERV-K HML-2 Env trimer (PDB: 9NND). Surface subunit (SU): green; transmembrane subunit (TM): purple. (**b**) Postfusion HERV-K HML-2 TM fragment from the Kenv-4-bound complex (antibody omitted), highlighting the refolded TM core, including HR1, HR2, and the tether helix (PDB: 9MLK). HR1: pink; HR2: purple; tether helix: yellow. (**c**) Structure of syncytin-1 RBD from the soluble G439-truncated construct bound to wild-type ASCT2 (PDB: 8OUH; 2.62 Å). Syncytin-1: cyan; ASCT2: grey. (**d**) Structure of suppressyn RBD bound to consensus-engineered ASCT2 (PDB: 8OUI; 3.39 Å). Suppressyn: raspberry red; ASCT2: grey. (**e**) Postfusion TM structure of syncytin-1, highlighting the N helix, C helix, CX_6_CC motif, and T loop (PDB: 6RX1). N helix: pink; C helix: purple; CX_6_CC motif and T loop: yellow. (**f**) Postfusion TM structure of syncytin-2, highlighting the N helix, C helix, CX_6_CC motif, and T loop (PDB: 6RX3). N helix: pink; C helix: purple; CX_6_CC motif and T loop: yellow. (**g**) Structure of the syncytin-2 SU–MFSD2A complex obtained using consensus-engineered proteins and the syncytin-2 C43S substitution (PDB: 7OIX; 3.6 Å). Syncytin-2: salmon; MFSD2A: grey. Curved arrows in panels (**a**–**d**) indicate 90° rotations.

Shaked et al. [95] determined a 2.1 Å cryo-EM structure of detergent-solubilised, full-length Phoenix consensus Env in a prefusion trimer. Phoenix is an experimentally reconstructed HML-2 sequence assembled from multiple human proviral copies, rather than the product of a single naturally occurring locus [42]. The trimer is elongated and relatively open, with separated SUs surrounding a central cavity (Figure 4a). SU extends towards the membrane-proximal region and contacts the TM stem. This organisation differs substantially from the compact lentiviral Env architecture, while retaining a recognisable fusion apparatus. The structure therefore supplies candidate surfaces for receptor and antibody studies. However, detergent extraction and consensus reconstruction limit direct extrapolation to locus-specific Envs in cellular membranes, and a static prefusion structure does not establish the sequence of receptor- or pH-dependent triggering events.

Shek et al. [96] used an engineered soluble Phoenix ectodomain to resolve prefusion Env and antibody-bound conformations. Although proline substitutions were screened, the successful prefusion construct combined an SU–TM disulphide, a modified furin-cleavage sequence and a T4 fibritin trimerisation domain. Conformation-sensitive antibodies also enabled resolution of a separately expressed postfusion TM fragment, revealing the additional tether helix (Figure 4b). These are complementary structural endpoints, not direct observations of a single trimer undergoing fusion. Agreement between the engineered ectodomain and the full-length detergent-solubilised structure supports the overall prefusion fold. It does not establish native conformational populations, triggering kinetics or the effects of locus-specific sequence and glycosylation differences. Antibody-bound structures define epitopes and provide useful probes, but binding or staining alone does not establish neutralisation or therapeutic efficacy.

A subsequent 2.25 Å crystal structure of isolated, consensus HERV-K SU adds an independent view of the receptor-facing subunit [97]. It identifies candidate interaction surfaces, including a basic groove and hydrophobic patches. These are hypotheses for functional testing: bound crystallisation components and surface geometry do not by themselves identify a physiological receptor or establish an effective inhibitor.

### 5.2. Domesticated Retroviral Envelope Remnants: Syncytin-1, Suppressyn, and Syncytin-2

Due to the multifunctional abilities of retroviral envelopes, including fusogenic and immunosuppressive properties, they have been co-opted multiple times in vertebrates to form new cellular genes. The best-characterised co-opted ERV envelopes are syncytin-1 (from a HERV-W *env*) and syncytin-2 (from a HERV-FRD *env*), which contribute to placental development in humans. Specifically, syncytins promote trophoblast cell–cell fusion and maintain the syncytiotrophoblast, a multinucleated epithelial layer that covers chorionic villi and forms the primary maternal-foetal exchange interface, supporting nutrient and gas transport, hormone production, and immune barrier functions [49,91,92,98]. Syncytin capture has occurred multiple independent times across mammalian evolution, contributing to the diversity of placentation strategies [99,100]. Sequence-based studies have identified a furin-cleavage motif and an Env-related immunosuppressive-domain region within syncytins [101,102]. More recently, structural studies have begun to translate these sequence features into a mechanistic understanding of receptor engagement and fusion control [91,92,98,103]. In the same context of human development, suppressyn, derived from a HERV-H *env* gene [103,104,105], acts as an anti-fusogenic regulator by antagonising the syncytin-1 receptor pathway. These proteins illustrate complementary roles proposed for Env-derived proteins in placental development, and structural studies have begun to uncover the molecular interactions involved.

Khare et al. [103] resolved syncytin-1 recognition of wild-type ASCT2, the trimeric neutral amino acid transporter encoded by *SLC1A5*, using a soluble syncytin-1 construct truncated at G439. A single receptor-binding domain occupies the extracellular vestibule of the ASCT2 trimer and forms a hybrid β-sheet clamp with a receptor hairpin (Figure 4c). The maps resolve the receptor-bound RBD rather than an intact Env trimer. The 2.62 Å reconstruction defines the binding interface, while a separate 3.5 Å reconstruction resolves the otherwise poorly ordered transport domain of the third ASCT2 subunit. Ruigrok et al. [92] characterised the syncytin-1 TM ectodomain in its postfusion 6HB state (Figure 4e), establishing conservation of the class I fusion endpoint. Together, these structures define receptor engagement and the fusion endpoint, but leave the intervening activation pathway unresolved.

In the same study, Khare et al. [103] resolved suppressyn bound to consensus-engineered ASCT2 at 3.39 Å (Figure 4d). Suppressyn lacks the TM fusion machinery but occupies an overlapping receptor site, forming two β-sheet clamps, one of which corresponds to the syncytin-1 clamp. The overlapping interfaces explain why the two proteins cannot bind the same ASCT2 trimer simultaneously. Purified suppressyn and the soluble syncytin-1 construct inhibited syncytin-1-mediated cell fusion at 10 µM and partially inhibited ASCT2-mediated alanine transport in proteoliposomes at 20 µM. These functional observations support receptor competition and effects on transport under the tested conditions; their magnitude in placental tissue remains unresolved. Comparisons with AlphaFold models of ASCT2-using retroviral RBDs, including that of the type D betaretrovirus SRV3, suggest a related mechanism of receptor interference. Those viral receptor-bound complexes were not experimentally determined, so extension of the clamp mechanism to them remains a structural prediction.

Syncytin-2 provides a second example of conserved fusion machinery following domestication. Renard et al. [91] solved a central ectodomain fragment with a fold similar to corresponding regions of infectious retroviral Envs despite substantial sequence divergence. Ruigrok et al. [92] subsequently resolved the postfusion TM 6HB, comprising a central trimeric coiled coil with antiparallel outer helices (Figure 4f). These structures support retention of the class I fusion mechanism, but the use of fragments limits conclusions about the complete prefusion assembly and how receptor binding activates it.

Martinez-Molledo et al. [98] determined a 3.6 Å cryo-EM structure of syncytin-2 SU bound to MFSD2A (Figure 4g). MFSD2A is a sodium-dependent lysophosphatidylcholine (LPC) transporter that supplies essential fatty acids to the brain and also serves as the syncytin-2 receptor. Thermostabilised consensus designs and a fusion-defective C43S substitution in syncytin-2 enabled structural analysis. Focused reconstruction resolved a 1:1 SU–receptor complex, with MFSD2A in an outward-facing occluded state; it did not resolve the complete SU–TM fusion assembly. Three SU receptor-binding loops contact extracellular regions of MFSD2A, and mutations in these loops reduced cell fusion, supporting the functional relevance of the interface. The captured conformation suggests that receptor binding could restrict transporter movements, while leaving the dynamics of native membrane-bound proteins unresolved.

In a separate cell-based transport assay, a purified, soluble consensus syncytin-2 SU fragment lacking the TM fusion machinery (SYNC2^CO^-SU) inhibited MFSD2A-mediated LPC uptake with an IC_50_ of approximately 200 nM [98]. HEK-293T.17 cells expressing consensus MFSD2A were preincubated with the fragment for 10 min at 37 °C, followed by a 10-min uptake assay using 20 µM fluorescent LPC analogue (LPC_NBD_). The fragment was produced as a secreted recombinant protein in HEK-293F cells. The study did not establish that an equivalent soluble species is physiologically released or reaches inhibitory concentrations in the relevant tissue. Thus, the assay demonstrates experimental transport inhibition, while a transport-regulatory role for endogenous syncytin-2 remains unproven.

These examples distinguish retention of ancestral molecular machinery from its deployment in a host process. They do not by themselves demonstrate that receptor specificity changed during domestication, because the corresponding ancestral receptor-bound structures are generally unavailable. Independent captures of placentally expressed Env genes in mice, guinea pigs, tenrecs, ruminants and carnivores [106,107,108,109,110] offer a comparative route to this question. Comparing their structures and receptor interactions could test whether similar placental functions repeatedly use conserved fusion machinery, lineage-specific recognition interfaces or both. Functional comparisons will be needed to connect those structural differences to placental biology.

## 6. Conclusions and Outlook

Structural studies are beginning to refine our understanding of endogenous retroviruses by linking their evolutionary history to molecular mechanisms. Some ERV proteins retain core features of their retroviral ancestors, whereas others have been substantially remodelled through domestication to support host functions.

For retroviral evolution, HERV-K and RELIK structures show that divergent or reconstructed proteins can retain recognisable folds and interaction surfaces. These observations constrain models of ancestral molecular function, but a consensus sequence is not a uniquely determined ancestor. Many ERV families remain structurally undersampled, and the relationship between reconstructed proteins and naturally encoded locus variants requires direct testing. Structure-guided comparisons are therefore most useful when integrated with sequence reconstruction, genomic context and experimentally tested alternatives. Conserved sites may suggest targets for chemical probes, but structural conservation alone does not establish druggability or broad-spectrum therapeutic potential.

For host biology, syncytins and suppressyn show how retained receptor-binding and fusion-related features can support distinct cellular functions. Establishing the contribution of a structural feature to domestication requires more than recognising a viral-like fold: perturbing that feature should alter a relevant host phenotype. Receptor-binding assays, cell-fusion experiments and studies in physiologically appropriate tissues are therefore essential links between structural interpretation and biological function.

Future progress will depend on connecting structures across scales and experimental contexts. Single-particle cryo-EM can resolve assemblies within intact virions and particles, while cryo-ET is particularly valuable for examining their organisation and interactions within cells. Prediction can guide construct design and comparisons across divergent ERV families, but uncertain interfaces, flexible regions and membrane-associated conformations require experimental validation. Combining these methods with locus-resolved expression analysis and functional perturbation should help distinguish proteins that are merely expressed from those that form active assemblies or contribute to host phenotypes. For BHIKHARI, a specific priority is to test whether the predicted CA-like proteins assemble and whether that assembly is required for their developmental functions.

ERV structural biology can therefore inform both the history of retroviral molecular mechanisms and the emergence of host functions. Its central challenge is to connect a defined sequence and structural state to a measurable biological effect. Addressing that challenge will require comparisons among reconstructed and naturally encoded proteins, and experiments that test proposed mechanisms in relevant cellular settings. This would provide a firmer basis for evaluating disease associations and future applications in therapeutic targeting or protein engineering.

## Data Availability

All sequences used in the phylogenetic analyses were obtained from public databases or previously published studies, with sequence details and sources listed in Supplementary Tables S1 and S2. The trimmed alignments and maximum-likelihood tree files underlying Figure 1 and Figure 2 are available from the corresponding authors on reasonable request. Accession codes and availability information for experimental structural data in the Protein Data Bank (PDB) and, where applicable, the Electron Microscopy Data Bank (EMDB) are provided in the cited original studies. PDB accession codes for the structures illustrated in Figure 3 and Figure 4 are also listed in the corresponding figure legends.

## Supplementary Materials

The following supporting information can be downloaded at: https://www.mdpi.com/article/10.3390/v18101132/s1, Table S1: Gag amino-acid sequences used in this study; Table S2: Env amino-acid sequences used in this study.
