# Supplementary material for: Structural Insights into Endogenous Retroviruses: Evolution and Co-Opted Functions
Source: Viruses. 2026 Oct 13;18(10):1132. doi: 10.3390/v18101132 (PMC13709493; doi:10.3390/v18101132)
Supplement: Supplementary file 1 [file viruses-18-01132-s001.zip › viruses-4564644-supplementary.pdf]

Supplementary Table S1. Gag amino-acid sequences used in this study

Number	Name	Length (aa)	Accession	Source
**1**	HERVL	1045	DF000000198.4	Dfam
**2**	pSIVconsensus	448	—	1
**3**	ERV-Gamma-Iba	683	—	
**4**	ERV-Epsilon-Npo	791	—	
**5**	ERV-Epsilon-Dti	793	—	
**6**	ERV-Epsilon-Rmi	608	—	
**7**	ERV-Epsilon-Lpi	471	—	
**8**	ERV-Spuma-Spu	435	—	2
**9**	ERV-Spuma-Tgr	402	—	
**10**	ERV-Spuma-Nde	544	—	
**11**	ERV-Spuma-Clu	523	—	
**12**	ERV-Epsilon-Hph	379	—	
**13**	ERV-Epsilon-Bsu	279	—	
**14**	HML1	549	hml1_20010_100020_con	
**15**	HML2	700	hml2_20020_100022_con	
**16**	HML3	707	hml3_20030_100023_con	
**17**	HML4	769	hml4_20040_100024_con	
**18**	HML5	518	hml5_20050_100025_con	
**19**	HML6	561	hml6_20060_100026_con	
**20**	HML7	625	hml7_20070_100027_con	
**21**	HML8	714	hml8_20080_100028_con	
**22**	HML9	624	hml9_20090_100029_con	
**23**	HERV9	875	herv9_10320_100006_con	
**24**	HERVW	503	hervw_10310_100019_con	3
**25**	HERVADP	624	hervadp_10420_100007_con	
**26**	HERV4	537	herv4_10760_100005_con	
**27**	HERV3	580	herv3_10230_100004_con	
**28**	HERVE	619	herve_10210_100008_con	
**29**	HERVT	506	hervt_10110_100018_con	
**30**	HERVFC	560	hervfc_10650_100011_con	
**31**	HERVH48	627	hervh48_10620_100013_con	
**32**	HERVFB	623	hervfb_10640_100010_con	
**33**	HERVFA	646	hervfa_10630_100009_con	
**34**	HERVFRD	420	hervfrd_10710_3633_bre	
**35**	HERVH	1375	hervh_10610_100012_con	
**36**	Desmodus rotundus endogenous retrovirus	728	MH648003.1	4
**37**	Miniopterus natalensis arenarius endogenous virus MINERVa	353	KY250075.1	5
**38**	Springhare endogenous lentivirus (SpELV)	453	—	6
**39**	Galeopterus variegatus endogenous lentivirus (GvaELV)	469	—	7

**40**	Porcine endogenous retrovirus C	524	ABL74441.1	GenBank
**41**	Rabbit endogenous lentivirus type K (RELIK)	477	AZY88392.1	GenBank
**42**	Small ruminant lentivirus (SRLVs)	444	AYG99193.1	GenBank
**43**	Puma lentivirus 14 (PLV-14)	479	YP_009507790.1	GenBank
**44**	Python molurus endogenous retrovirus (PyERV)	1637	AAN77283.1	GenBank
**45**	Feline immunodeficiency virus (FIV)	450	NP_040972.1	GenBank
**46**	Moloney murine leukemia virus (Mo-MLV)	538	AAL69909.1	GenBank
**47**	Feline leukemia virus (FeLV)	580	AAA43054.1	GenBank
**48**	Gibbon ape leukemia virus (GaLV)	523	ALV83308.1	GenBank
**49**	Mouse mammary tumor virus (MMTV)	591	NP_056882.1	GenBank
**50**	Jaagsiekte sheep retrovirus (JSRV)	612	NP_041184.1	GenBank
**51**	Simian retrovirus 1 (SRV)	658	AAA47730.1	GenBank
**52**	Human T-cell leukemia virus type I (HTLV-1)	429	NP_057862.1	GenBank
**53**	Bovine leukemia virus (BLV)	393	NP_056897.1	GenBank
**54**	Bovine foamy virus (BFV)	544	AAB68769.1	GenBank
**55**	Feline foamy virus (FFV)	514	YP_009513248.1	GenBank
**56**	Human immunodeficiency virus 1	508	AAD39400.1	GenBank
**57**	human endogenous retrovirus type K (HERV-Kcon)	666	—	8
**58**	BHIKHARI	616	CAB51074.1	GenBank
**59**	BHIKHARI-2	593	NP_571068.2	GenBank

References

1 Gilbert, C., Maxfield, D. G., Goodman, S. M. & Feschotte, C. Parallel Germline Infiltration of a Lentivirus in Two Malagasy Lemurs. *PLOS Genetics* **5**, e1000425 (2009). https://doi.org/10.1371/journal.pgen.1000425

2 Harding, E. F., Webster, B., Sridhar, T., Mercer, L. K. & White, P. A. Transcriptome mining reveals diversity and evolution of circulating and endogenous amphibian retroviruses. *Retrovirology* **22**, 13 (2025). https://doi.org/10.1186/s12977-025-00669-y

3 Vargiu, L. *et al.* Classification and characterization of human endogenous retroviruses; mosaic forms are common. *Retrovirology* **13**, 7 (2016). https://doi.org/10.1186/s12977-015-0232-y

4 Franco Filho, L. C. *et al.* Complete Endogenous Retrovirus Genome Sequence from a Brazilian Vampire Bat (Desmodus rotundus). *Microbiol Resour Announc* **8** (2019). https://doi.org/10.1128/mra.01497-18

5 Farkašová, H. *et al.* Discovery of an endogenous Deltaretrovirus in the genome of long-fingered bats (Chiroptera: Miniopteridae). *Proceedings of the National Academy of Sciences* **114**, 3145-3150 (2017). https://doi.org/doi:10.1073/pnas.1621224114

6 Kambol, R., Gatseva, A. & Gifford, R. J. An endogenous lentivirus in the germline of a rodent. *Retrovirology* **19**, 30 (2022). https://doi.org/10.1186/s12977-022-00615-2

7 Han, G.-Z. & Worobey, M. A Primitive Endogenous Lentivirus in a Colugo: Insights into the Early Evolution of Lentiviruses. *Molecular Biology and Evolution* **32**, 211-215 (2015). https://doi.org/10.1093/molbev/msu297

8 Lee, Y. N. & Bieniasz, P. D. Reconstitution of an Infectious Human Endogenous Retrovirus. *PLOS Pathogens* **3**, e10 (2007). https://doi.org/10.1371/journal.ppat.0030010

Supplementary Table S2. Env amino-acid sequences used in this study

Number	Name	Length (aa)	Accession	Source
**1**	HERVL32	234	hervl32_30220_1946_bre	
**2**	HERVS	463	hervs_30200_100017_con	
**3**	HML2	715	hml2_20020_100022_con	
**4**	HML4	699	hml4_20040_100024_con	
**5**	HML5	665	hml5_20050_100025_con	
**6**	HML6	678	hml6_20060_100026_con	
**7**	HML7	742	hml7_20070_100027_con	
**8**	HML8	853	hml8_20080_100028_con	
**9**	HML9	658	hml9_20090_100029_con	
**10**	HML10	625	hml10_20100_100021_con	
**11**	HERVH48	597	hervh48_10620_100013_con	
**12**	HERVH	691	hervh_10610_100012_con	1
**13**	HERVFC	544	hervfc_10650_100011_con	
**14**	HERVFB	542	hervfb_10640_100010_con	
**15**	HERVFA	537	hervfa_10630_100009_con	
**16**	HERV30	551	herv30_10330_2924_bre	
**17**	HERV9	670	herv9_10320_100006_con	
**18**	HERV4	621	herv4_10760_100005_con	
**19**	HERV1	786	herv1_10240_100002_con	
**20**	HERV3	707	herv3_10230_100004_con	
**21**	HERVE	695	herve_10210_100008_con	
**22**	HERVT	632	hervt_10110_100018_con	
**23**	HERVW / Syncytin-1	542	hervw_10310_100019_con	
**24**	HERVFRD / Syncytin-2	538	P60508	UniProt
**25**	Human immunodeficiency virus 1 (HIV-1)	859	AAL01570.1	GenBank
**26**	Gibbon ape leukemia virus (GaLV)	685	ALV83310.1	GenBank
**27**	Murine leukemia virus (MLV)	665	AAA46526.1	GenBank
**28**	Jaagsiekte sheep retrovirus (JSRV)	615	CAA01902.1	GenBank
**29**	Mouse mammary tumor virus (MMTV)	688	NP_056883.1	GenBank
**30**	pSIVconsensus	708	—	2
**31**	HERV-Kcon	698	—	3

References

1 Vargiu, L. *et al.* Classification and characterization of human endogenous retroviruses; mosaic forms are common. *Retrovirology* **13**, 7 (2016). https://doi.org/10.1186/s12977-015-0232-y

2 Gilbert, C., Maxfield, D. G., Goodman, S. M. & Feschotte, C. Parallel Germline Infiltration of a Lentivirus in Two Malagasy Lemurs. *PLOS Genetics* **5**, e1000425 (2009). https://doi.org/10.1371/journal.pgen.1000425

3 Lee, Y. N. & Bieniasz, P. D. Reconstitution of an Infectious Human Endogenous Retrovirus. *PLOS Pathogens* **3**, e10 (2007). https://doi.org/10.1371/journal.ppat.0030010
